# Transcriptomic Insights into Paclobutrazol-Induced Modulation of Metabolic and Signaling Pathways During Microtuberization of Potato *Solanum tuberosum* L.

**DOI:** 10.3390/ijms27104618

**Published:** 2026-05-21

**Authors:** Lisset Herrera-Isidron, Andrea María Navarro-Vega, Braulio Uribe-López, Ilse Araceli Careaga-Rojas, Danae Carrillo-Ocampo, Aaron Barraza, Eliana Valencia-Lozano, José Luis Cabrera-Ponce

**Affiliations:** 1 Unidad Profesional Interdisciplinaria de Ingeniería Campus Guanajuato (UPIIG), Instituto Politécnico Nacional, Av. Mineral de Valenciana 200, Puerto Interior, Silao de la Victoria 36275, Mexico; lherrerai@ipn.mx (L.H.-I.); anavarrov1900@alumno.ipn.mx (A.M.N.-V.); icareagar2000@alumno.ipn.mx (I.A.C.-R.); 2 Departamento de Biotecnología y Bioquímica, Centro de Investigación y de Estudios Avanzados del IPN, Unidad Irapuato, Irapuato 36824, Mexico; braulio.uribe@cinvestav.mx; 3 Laboratorio Nacional PlanTECC, Laboratorio de Cromatografía, Departamento de Ingeniería Genética, Centro de Investigación y de Estudios Avanzados del IPN, Unidad Irapuato, Irapuato 36824, Mexico; danae.carrillo@cinvestav.mx; 4 Laboratorio Biotechnolgika, Ignacio Ramirez 3765, La Paz 23060, Mexico; abarrazc@gmail.com; 5 Laboratorio de Investigación Interdisciplinaria (LII), Universidad Nacional Autónoma de México, Escuela Nacional de Estudios Superiores, Unidad León, León de los Aldama 37684, Mexico; 6 Laboratorio Nacional PlanTECC, Laboratorio de Modificación Genética, Departamento de Ingeniería Genética, Centro de Investigación y de Estudios Avanzados del IPN, Unidad Irapuato, Irapuato 36824, Mexico

**Keywords:** paclobutrazol, MTs development, gibberellins, *ATH1*, *Solanum tuberosum* L., H_2_S signaling

## Abstract

Paclobutrazol (PBZ) is a triazole-type plant growth regulator that interferes with gibberellin (GAs) biosynthesis by blocking the oxidation step that converts ent-kaurene into ent-kaurenoic acid; however, the developmental mechanisms linking GAs restriction with storage organ enlargement remain poorly understood. In potato, PBZ induces compact growth while promoting microtubers (MTs) expansion, suggesting that GAs depletion triggers coordinated developmental reprogramming rather than simply suppressing elongation. Here, we evaluated the phenotypic, histological, and transcriptomic responses associated with PBZ-induced MTs development in *Solanum tuberosum* L. PBZ treatment, which increased MTs size, suppressed stolon growth, and enhanced starch accumulation, indicating a shift toward storage tissue development. Transcriptomic analysis identified broad PBZ-responsive changes, including enrichment of pathways related to metabolism, ribosome function, carbon metabolism, plant hormone signaling, and cell cycle regulation. Network analyses revealed ATH1-associated modules connected with receptor-like kinases, transcriptional regulators, mitotic regulators, replication-licensing factors and condensin components, supporting coordinated regulation among growth control, localized proliferation, asymmetric division, endoreduplication, and chromatin stability. These patterns were further supported by the absence of a detectable gibberellic acid (GA_3_) peak in PBZ-treated samples. These findings support a model in which PBZ-responsive signaling is associated with developmental reprogramming toward radial expansion and reinforcement of storage tissue, providing a regulatory mechanism by which growth repression is coupled to microtube enlargement in potato.

## 1. Introduction

Potato, *S. tuberosum* L., is the fourth most important food crop worldwide and a critical resource for global food security [1]. It has long been valued for its ability to thrive under extreme environmental conditions [2].

MTs development has become an essential tool for standardized seed production and for dissecting the physiological and molecular mechanisms that regulate tuber development [3,4,5].

Recent studies have further refined the molecular understanding of potato tuberization, revealing that MTs development is governed by highly integrated regulatory networks involving phytohormone signaling, carbon allocation, and stress-responsive transcriptional programs [6,7]. Recent transcriptomic and multi-omics analyses of potato tuberization have additionally identified candidate genes and metabolic pathways associated with storage organ differentiation, carbohydrate metabolism, hormonal regulation, and developmental reprogramming during tuber formation, reinforcing the complexity of tuberization processes in *S. tuberosum* L. [8,9,10].

The regulation of tuber development and quality is strongly influenced by phyto-hormones. Abscisic acid (ABA), GAs, cytokinins (CKs), auxins, and ethylene (ETH) act as key signaling molecules during tuber formation. In addition, brassinosteroids (BRs) are important regulators of potato tuber sprouting, while jasmonates (JAs), strigolactones (SLs), and salicylic acid (SA) also contribute to the control of dormancy and sprouting [11].

During the late 1960s, a group of compounds classified as 1-substituted imidazoles and 1,2,4-triazoles were introduced commercially and rapidly became effective agents for controlling fungal infections in both agricultural and medical contexts [12].

Over time, it became evident that these azole compounds also impact plant physiology by interfering with internal metabolic pathways, particularly those linked to terpenoid-derived hormones such as GAs and ABA [13,14].

In potato, a crop whose productivity relies heavily on hormonal regulation of tuber formation, azoles have been associated with changes in plant architecture, improved stress tolerance, and shifts in assimilate distribution, supporting their potential role in enhancing yield and tuber quality [15,16,17].

Among these compounds, PBZ, a triazole-based plant growth regulator, [(2RS,3RS)-1-(4-chlorophenyl)-4,4-dimethyl-2-(1H-1,2,4-trizol-1-yl)-pentan-3-ol], acts as a potent inhibitor of GAs biosynthesis through the blockade of *CYP701A*-mediated ent-kaurene oxidation. Its triazole moiety coordinates with the heme iron of cytochrome P450 monooxygenases, interfering with oxygen activation during the catalytic cycle and thereby inhibiting GAs biosynthesis [13].

Under low GAs conditions, such as those generated by PBZ treatment, DELLA proteins become stabilized and accumulate. DELLA proteins function as central repressors of plant growth and interact with multiple transcriptional regulators to modulate developmental programs [18,19].

Among these transcriptional regulatory networks that are increasingly recognized as central integrators of developmental, hormonal, and stress-responsive pathways, enabling plants to coordinate adaptive growth responses under changing environmental conditions, the homeobox transcription factor (TF) *ATH1* plays a key role in controlling plant architecture by locally restricting growth responses. ATH1 and DELLA proteins act cooperatively to suppress elongation growth, integrating hormonal signaling with transcriptional programs that regulate meristem activity and organ architecture [20,21].

Inhibition of GAs biosynthesis may also alter metabolic flux through isoprenoid intermediates such as geranylgeranyl pyrophosphate (GGPP), favoring their redirection toward the ABA-related pathway [13]. Increased ABA accumulation has been associated with the activation of hydrogen sulfide (H_2_S) signaling, an important component of plant stress responses. ABA perception through PYR/PYL/RCAR receptors initiates a signaling cascade that can promote the expression of L-cysteine desulfhydrase (*DES1*), a major enzymatic source of endogenous H_2_S. In turn, H_2_S has been shown to modulate ABA signaling through protein persulfidation, thereby contributing to stomatal regulation and stress tolerance [22].

PBZ enhances the antioxidant capacity of plants by increasing the activities of enzymes such as superoxide dismutase (SOD), catalase (CAT) and peroxidase (POD), while also promoting the accumulation of soluble proteins and chlorophyll [16,23,24,25].

These changes collectively contribute to improved redox homeostasis and greater tolerance to abiotic stress. Although PBZ typically reduces overall plant biomass, it promotes higher tuber yield by redirecting a greater proportion of assimilates toward tuber development, thereby improving both the quantity and quality of tubers produced under challenging environmental conditions [17].

Overall, the molecular and physiological effects of PBZ on potato development provides key insights into the regulation of tuber formation. This knowledge contributes to improving MTs production systems and supports the development of strategies to enhance yield, quality, and stress resilience, ultimately promoting more sustainable crop production.

## 2. Results

### 2.1. Effects of PBZ on MTs Development

**Figure 1:**
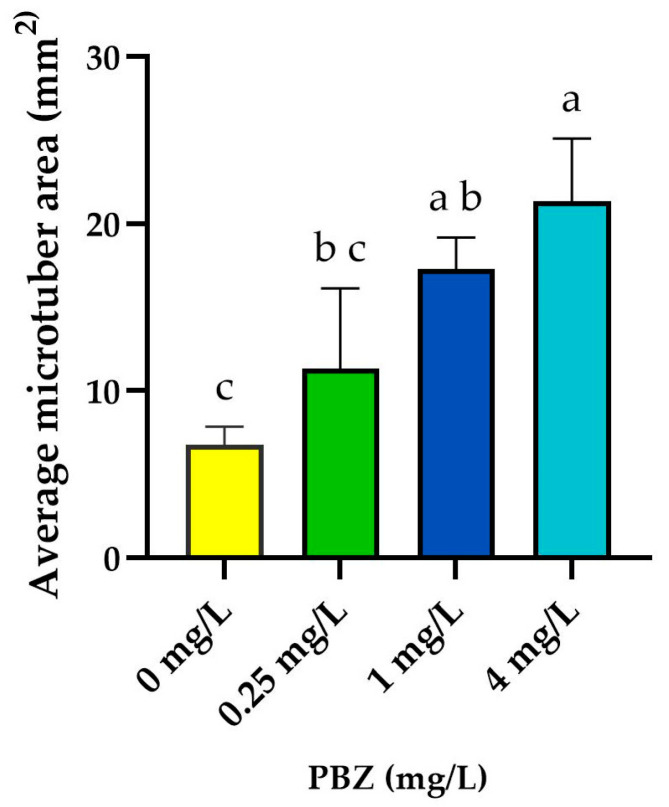
Effect of PBZ on potato MTs area. Data are presented as the mean ± SD from three independent biological replicates. Different letters indicate statistically significant differences among treatments according to one-way ANOVA followed by Tukey’s multiple comparison test (*p* < 0.05).

MTs development from *S. tuberosum* L. cv. Alpha was significantly influenced by PBZ concentration. The microtuber area increased in a dose-dependent manner with increasing PBZ levels, showing a marked rise between 0 and 1 mg/L, followed by a more gradual increase up to 4 mg/L (Figure 1). The highest MTs enlargement was observed at 4 mg/L, reaching an average area of 21.37 mm^2^.

Statistical analysis showed that the 1 and 4 mg/L PBZ treatments produced significantly larger MTs areas compared with the control, whereas the 0.25 mg/L treatment did not differ significantly from the control. No significant differences were detected between the 1 and 4 mg/L PBZ treatments.

**Figure 2:**
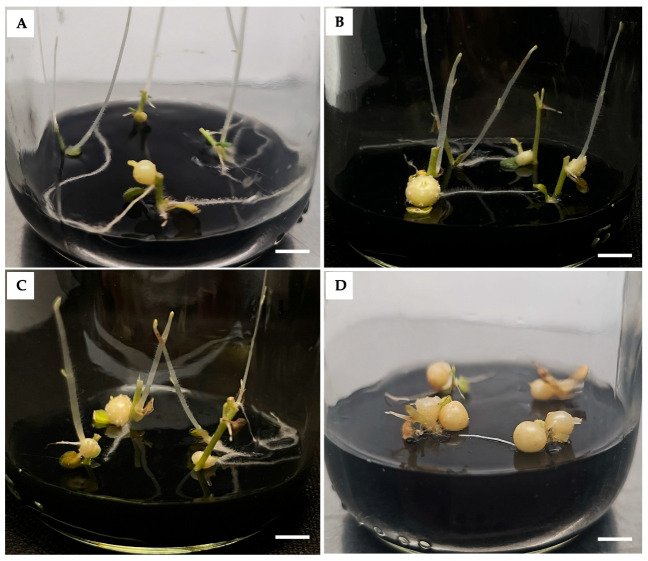
MTs obtained from *S. tuberosum* L. cv. Alpha cultured on MR8G62iP medium supplemented with PBZ: (**A**) 0 mg/L (control), (**B**) 0.25 mg/L, (**C**) 1 mg/L, and (**D**) 4 mg/L. Scale bar = 1 cm.

These results indicate a dose-dependent effect of PBZ on MTs growth. Additionally, morphological evaluation revealed that while stolon formation was evident in the control and lower PBZ concentrations, it was suppressed at 4 mg/L (Figure 2D).

### 2.2. Histological Changes During MTs Development

**Figure 3:**
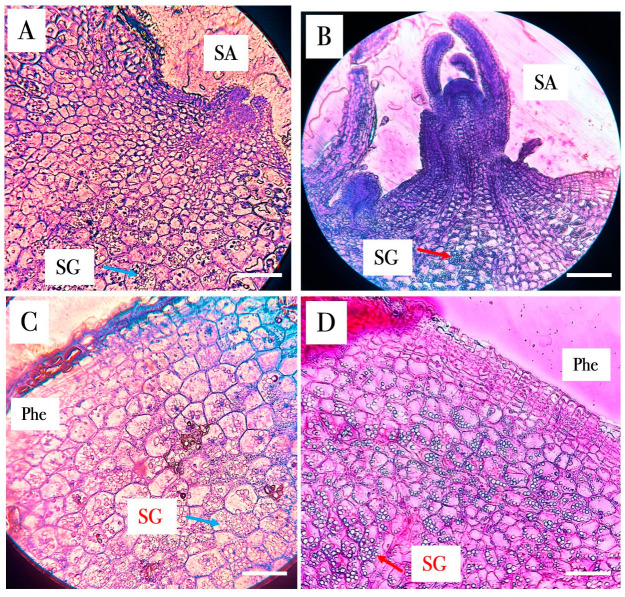
Histological analysis of MTs developed in control and PBZ (4 mg/L). (**A**,**C**). Control MTs, (**B**,**D**), PBZ-derived MTs. SA: Shoot apex, SG: Starch granules, Phe: Phellogen. Scale bar = 1 mm.

Histological analysis revealed that PBZ-treated MTs exhibited a more compact parenchyma organization, with reduced intercellular spaces and increased cellular density compared to control tissues (Figure 3). The phloem region appeared more distinctly organized, showing clearer vascular differentiation and a more defined spatial separation from surrounding storage parenchyma (Figure 3). These structural changes were accompanied by increased starch granule accumulation within parenchyma cells (Figure 3).

Quantification performed in 112 analyzed cells per treatment showed that PBZ-treated samples contained significantly more starch granules per cell (12.49 ± 9.41) than control samples (2.79 ± 3.35) according to Welch’s *t*-test (*p* < 0.0001). The quantification data are presented in the Supplementary Materials (Figure S1). These results suggest enhanced carbon allocation toward storage compounds under PBZ treatment.

### 2.3. Phenotypic Differences Between Control and PBZ-Derived Plants

**Figure 4:**
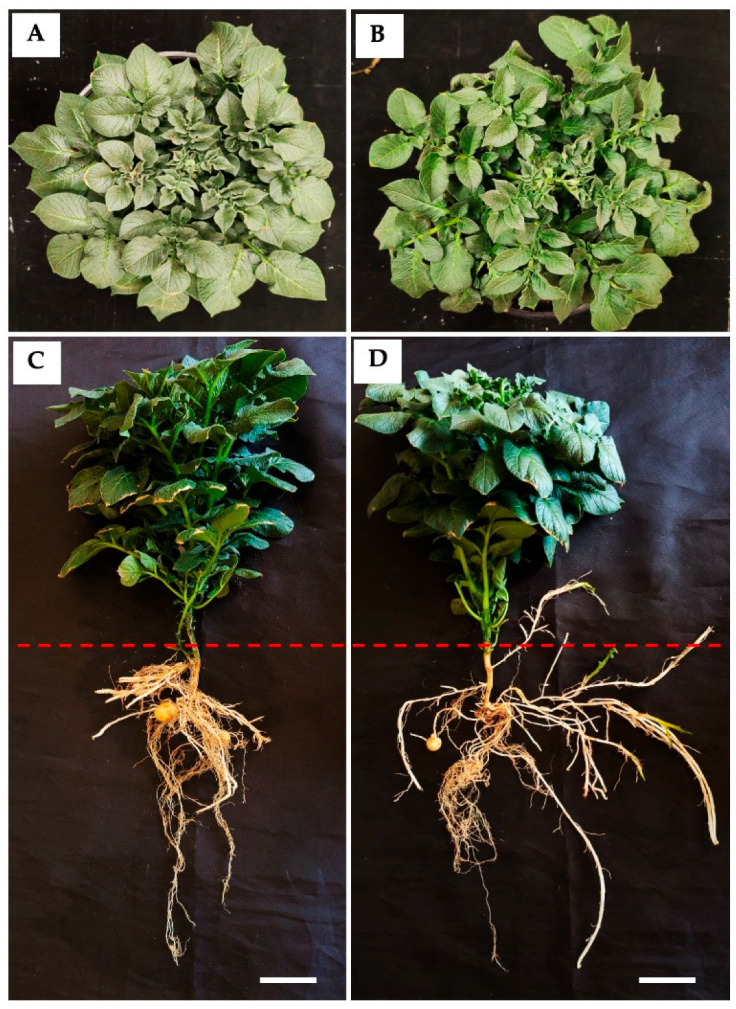
Phenotypic comparison of control (**A**,**C**) and PBZ (4 mg/L)-regenerated potato plants from MTs derived from PBZ treatment (**B**,**D**). (**A**,**B**) Shoot morphology showing differences in plant architecture and leaf area. (**C**,**D**) Whole plants illustrating root system architecture. PBZ-treated plants exhibit reduced shoot elongation, decreased leaf area, and enhanced root development relative to the control. The dashed red line indicates the boundary between the below-substrate and aerial tissue regions. Plants were grown in soil under controlled growth chamber conditions for 4 months. Scale bar = 1 cm.

Plants regenerated from PBZ-induced MTs exhibited a compact shoot architecture characterized by reduced stem elongation and decreased leaf area relative to controls. In contrast, PBZ-derived plants developed a more robust root system, with increased root expansion and a higher number of stolons (Figure 4).

Quantitative analysis supported the observed phenotypic differences between treatments (Table 1). PBZ-treated plants exhibited a more compact growth phenotype characterized by reduced stem length (20 ± 0.232 cm) and root length (20.71 ± 0.99 cm) compared with control plants (22.7 ± 2.78 cm and 28.03 ± 9.86 cm, respectively). Despite this reduction in elongation growth, PBZ treatment promoted traits associated with enhanced tuberization potential and plant vigor, including a greater stem width (1.285 ± 0.488 cm vs. 1.186 ± 0.286 cm in controls), a slightly greater root area (103.4 ± 1.87 cm^2^ vs. 102.2 ± 29.62 cm^2^), and a notably increased number of stolons (12 ± 1.41 vs. 8 ± 0.57). The leaf area was smaller in PBZ-treated plants (197.2 ± 0.11 cm^2^) compared with control plants (260.4 ± 27.65 cm^2^), consistent with the growth-retarding effect commonly associated with PBZ treatment. Overall, these results suggest that PBZ modulates plant architecture by limiting vegetative elongation while promoting morphological traits linked to stolon development and tuberization efficiency.

### 2.4. PBZ Transcriptomic-Wide Analysis

For transcriptomic analysis, total RNA was extracted from MTs tissues derived from stolon explants cultured under PBZ treatment. Samples were collected after 15 days of incubation in darkness, corresponding to an early stage of MTs development.

A total of 22,486 transcripts were detected in the RNA-seq dataset following filtering and mapping. Differential expression analysis identified 1916 significantly differentially expressed genes (DEGs) between PBZ-treated MTs (MR8G62iP supplemented with 4 mg/L of PBZ) and the control condition (MR8G62iP), of which 1447 genes were up-regulated and 469 were down-regulated according to the thresholds *p* < 0.05 and |log2FoldChange| ≥ 0.5.

**Figure 5:**
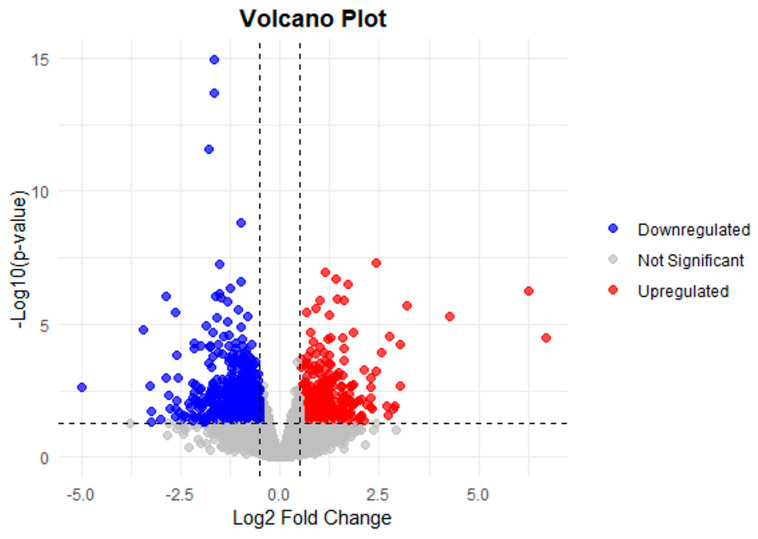
Volcano plot of differentially expressed genes (DEGs) associated with MTs induction under PBZ (4 mg/L). Genes were distributed according to |log2FoldChange| and statistical significance (−log10 *p*-value). Genes with *p* < 0.05 and |log2FoldChange| ≥ 0.5 were considered significantly differentially expressed. Up-regulated genes are shown on the right (red), whereas down-regulated genes are shown on the left (blue). Genes with higher statistical significance appear toward the top of the plot.

Most genes were distributed around log2FoldChange values close to zero, indicating limited transcriptional variation across the dataset. However, several genes exceeded the defined thresholds, suggesting moderate changes in gene expression associated with the experimental condition (Figure 5).

**Figure 6:**
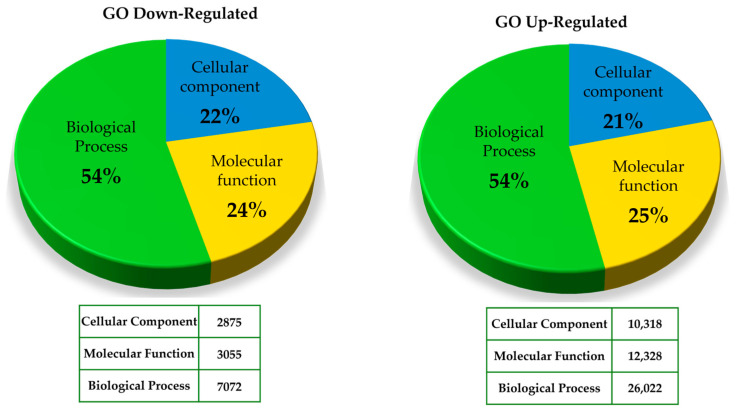
Gene Ontology (GO) classification of up- and down-regulated genes during potato *S. tuberosum* L. cv Alpha MTs development under PBZ (4mg/L) treatment.

A global overview of GO classification revealed that biological processes accounted for the largest proportion of both up-regulated and down-regulated genes (~54%), followed by molecular function (~24–25%) and cellular component (~21–22%) categories (Figure 6).

**Figure 7:**
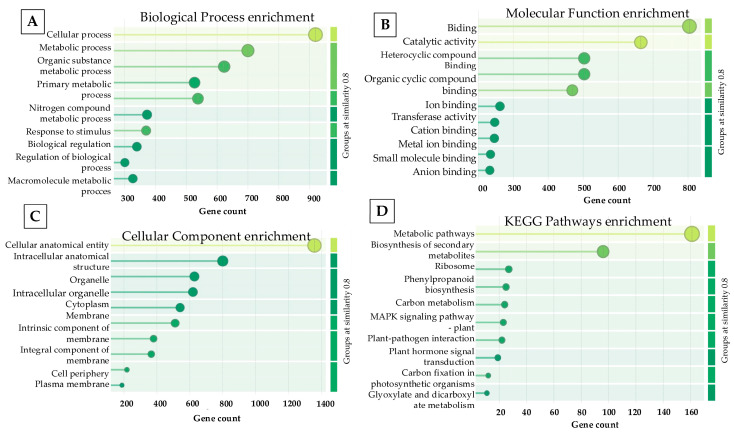
GO and KEGG enrichment analyses of differentially expressed genes during MTs development under PBZ (4mg/L) treatment. Biological process enrichment (**A**); Molecular function enrichment **(B**); Cellular component enrichment (**C**) and KEGG Pathways enrichment (**D**).

A Gene Ontology (GO) enrichment analysis of up-regulated genes revealed a total of 26,022 significantly enriched terms within the biological process category. The predominant terms included GO:0009987 (cellular process; 919), GO:0008152 (metabolic process; 700), and GO:0071704 (organic substance metabolic process; 623). Additional enriched categories comprised GO:0044237 (cellular metabolic process; 538) and GO:0044238 (primary metabolic process; 527), indicating the predominance of fundamental metabolic activities (Figure 7A).

GO enrichment analysis of down-regulated genes revealed a distinct functional profile compared to up-regulated genes, with enrichment in categories associated with growth-related processes, including cellular organization, developmental processes, and hormone-responsive pathways. In particular, several terms related to cell expansion, structural organization, and active growth regulation were significantly represented among down-regulated genes, suggesting suppression of elongation-associated and high-energy-demand biological functions under PBZ treatment.

Moreover, GO:0006807 (nitrogen compound metabolic process; 373) was also significantly enriched. Collectively, these results show that the enriched GO terms are mainly associated with cellular and metabolic processes (Figure 7A).

For molecular function, 12,328 significantly enriched GO terms were identified. The leading categories included GO:0005488 (binding; 804) and GO:0003824 (catalytic activity; 665), indicating a high proportion of proteins involved in molecular interactions and enzymatic functions. Additionally, GO:0097159 (organic cyclic compound binding; 503) and GO:1901363 (heterocyclic compound binding; 503) were enriched, reflecting the interaction of gene products with structurally complex organic molecules.

Furthermore, GO:0043167 (ion binding; 469) was also significantly represented, suggesting the participation of proteins in ion coordination. Overall, these findings indicate the prevalence of binding and catalytic activities among the analyzed gene products (Figure 7B).

Regarding cellular components, a total of 10,318 significantly enriched GO terms were identified. The dominant categories included GO:0110165 (cellular anatomical entity; 1357) and GO:0005622 (intracellular anatomical structure; 800), indicating that a large proportion of gene products are associated with intracellular structures. Additional enriched terms comprised GO:0043226 (organelle; 628), GO:0043229 (intracellular organelle; 619), and GO:0043227 (membrane-bounded organelle; 559), highlighting the localization of proteins within defined subcellular compartments.

Overall, the cellular component analysis indicates that the identified gene products are predominantly localized within intracellular and membrane-bound organelles (Figure 7C).

In the KEGG pathway category, the most significantly enriched pathways comprised metabolic pathways; the biosynthesis of secondary metabolites; ribosome, phenylpropanoid biosynthesis, carbon metabolism, MAPK signaling pathway, plant-pathogen interaction, plant hormones signal transduction, carbon fixation, glyoxylate and dicarboxylate metabolism were prominently represented (Figure 7D).

Enrichment significance was assessed using FDR-adjusted *p*-values (FDR < 0.05), and only pathways meeting this threshold were considered significantly enriched, indicating a robust transcriptional response to PBZ treatment.

#### 2.4.1. Up-Regulated Transcription Factors (TFs) Under PBZ Treatment

**Figure 8:**
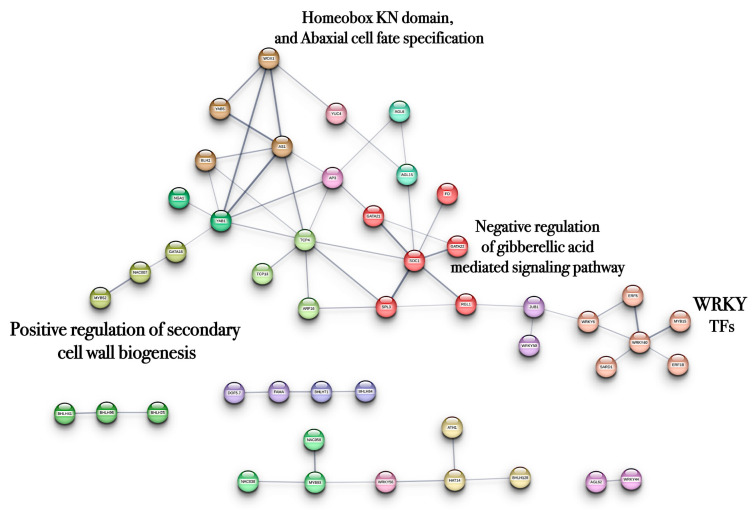
PPI network of up-regulated TFs involved in MTs development using PBZ (4 mg/L) from the transcriptomic-wide analysis with high confidence (0.500). Modules are highlighted with the name of the function. The figure represents a full network.

A protein–protein interaction (PPI) network was constructed using TF genes up-regulated in response to PBZ treatment based on their homologs in *Arabidopsis thaliana*. The network was further organized into distinct clusters according to functional associations (Figure 8).

Cluster analysis revealed groups of genes associated with the negative regulation of GA_3_ signaling (6), positive regulation of secondary cell wall biogenesis (3), and abaxial cell fate specification, including TFs containing homeobox KN domains (4). In addition, a cluster enriched in WRKY TFs was identified (6). The up-regulated TFs are listed in the Supplementary Materials (Table S1).

#### 2.4.2. Up-Regulated Genes Under PBZ Treatment

**Figure 9:**
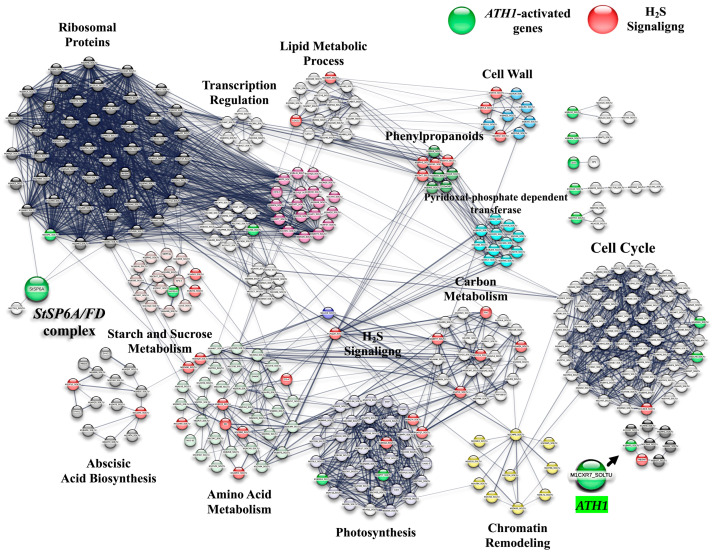
PPI network of up-regulated genes induced by PBZ. The network was made using STRING database of *potato S. tuberosum* L. cv. Alpha from the transcriptomic-wide analysis with high confidence (0.500). Clusters are highlighted with the name of the function. The figure represents a full network.

From 1447 up-regulated genes present in the PPI network with confidence (0.500) 349 were found to interact. In the PPI network, the following modules were observed: cell cycle (60), ribosomal proteins (48), amino acid metabolism (37), photosynthesis (36), carbon metabolism (22), PRE gene family: polyketide cyclase/dehydrase/lipid transporter (19), lipid metabolic process (16), starch and sucrose metabolism (16), isoprenoid/sterol/ABA biosynthesis (14), pyridoxal phosphate-dependent transferase (11), chromatin remodeling (10), cell wall/polysaccharide metabolism (9), phenylpropanoids, flavonoid biosynthesis (9), and leucine-rich repeat/serine/threonine protein kinase (8) (Figure 9).

The heat map displaying the up-regulated genes involved in PBZ treatment during potato development in darkness shows expression levels represented as log2 values. Genes were grouped according to their association with *ATH1*- and H_2_S-related pathways.

**Figure 10:**
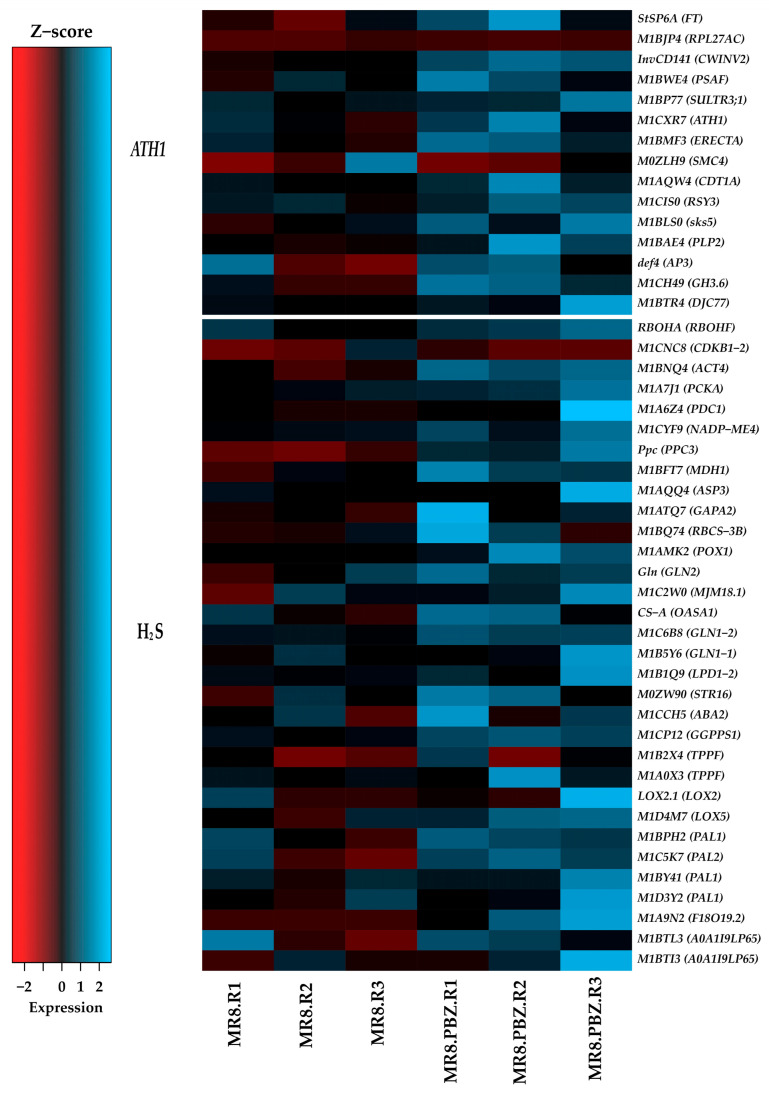
Heat map displaying the up-regulated genes induced by PBZ (4 mg/L) during potato MTs development in darkness; the up-regulation levels are shown in Log2.

Distinct expression patterns were observed across treatments. Genes associated with *ATH1* and H_2_S exhibited increased expression levels under PBZ treatment compared to control conditions, with consistent up-regulation across biological replicates (Figure 10). The up-regulated genes are listed in the Supplementary Materials (Table S2).

#### 2.4.3. Validation of DEGs by qPCR of PBZ Treatment (Relative Expression qPCR)

Validation of the transcriptome-wide analysis was performed by selecting eight differentially expressed genes (DEGs) and evaluating their expression levels by quantitative reverse transcription PCR (RT-qPCR) using the primers listed in the Supplementary Materials (Table S1). Gene expression was assessed after 15 days of incubation in darkness by comparing the control condition (MR8G62iP) and MR8G62iP supplemented with 4 mg/L of PBZ. Two endogenous reference genes were included for normalization. To evaluate the concordance between RNA-seq and RT-qPCR data, Pearson correlation analysis was performed using log2 fold-change values obtained from both datasets. The coefficient of determination (R^2^) was calculated to quantify the strength of the relationship between the two methods.

Genes were specifically selected based on PBZ treatment; these genes suggest relevance to the processes that respond to PBZ during MTs development.

Notably, *GGPP* (*M1CP12*), *ABA2* (*M1CCH5*), *OASA1* (*CS-A*) and *ASP3* (*M1AQQ4*) showed increased expression under PBZ treatment, consistent with previous reports linking these genes to hydrogen sulfide (H_2_S)-mediated responses. Additionally, *ERECTA* (*M1BMF3*) expression patterns were consistent with those of *ATH1* (*M1CXR7*), suggesting a coordinated role in developmental regulation. The remaining genes, including *SMC4* (*M0ZLH9*) and *CDT1a* (*M1AQW4*), were associated with cell cycle-related processes, indicating a potential involvement of PBZ in the regulation of cellular proliferation during MTs development.

**Figure 11:**
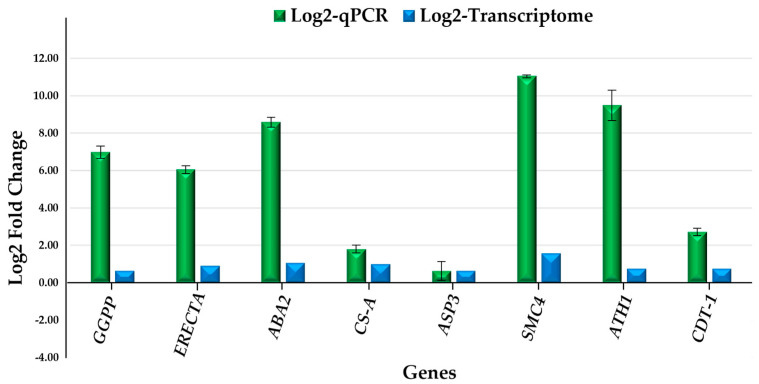
Comparison of gene expression in response to PBZ determined by qPCR and transcriptomic analysis. qPCR values are expressed as log2(^ΔΔCt^) for selected genes (*GGP*, *ERECTA*, *ABA2*, *CS-A*, *ASP3*, *SMC4*, *ATH1*, and *CDT1*), whereas RNA-seq data are presented as log2 fold change. Error bars in qPCR represent the SD ± 0.2 calculated from independent biological replicates (*n* = 3).

RT-qPCR results were consistent with transcriptomic data, showing similar expression trends across conditions. Gene expression was expressed as log2 fold change (log2FC; calculated as −ΔΔCt). Under PBZ treatment, all analyzed genes exhibited positive log2FC values, indicating up-regulation relative to the control condition. Notably, *SMC4* (11.04), *ATH1* (9.49), and *ABA2* (8.59) showed the strongest transcriptional responses, followed by *GGPP* (6.98) and *ERECTA* (6.06). Moderate changes were observed for *CDT-1* (2.72) and *CS-A* (1.81), while *ASP3* (0.63) displayed a relatively mild increase in expression (Figure 11).

To further evaluate the consistency between RNA-seq and RT-qPCR expression profiles, a Pearson correlation analysis was performed using the eight selected genes. A strong positive correlation was observed between both methodologies, with a Pearson correlation coefficient of r = 0.93 and a coefficient of determination of (R^2^) = 0.86, supporting the reliability of the transcriptomic expression trends validated by RT-qPCR (Figure 11).

Overall, PBZ treatment induced a coordinated transcriptional response, supporting the trends identified in the RNA-seq analysis.

#### 2.4.4. High-Performance Liquid Chromatography (HPLC) of GA_3_

The chromatographic absorbance profiles revealed pronounced differences among treatments. The GA_3_ standard exhibited a well-resolved peak at approximately 14 min, establishing the reference retention time for GAs detection under the experimental conditions.

The control sample, MTs without PBZ, displayed multiple chromatographic signals, including peaks in proximity to this retention time, suggesting the presence of endogenous GAs-like compounds. Collectively, these signals are indicative of a complex hormonal milieu, consistent with active GAs biosynthetic activity under control conditions.

**Figure 12:**
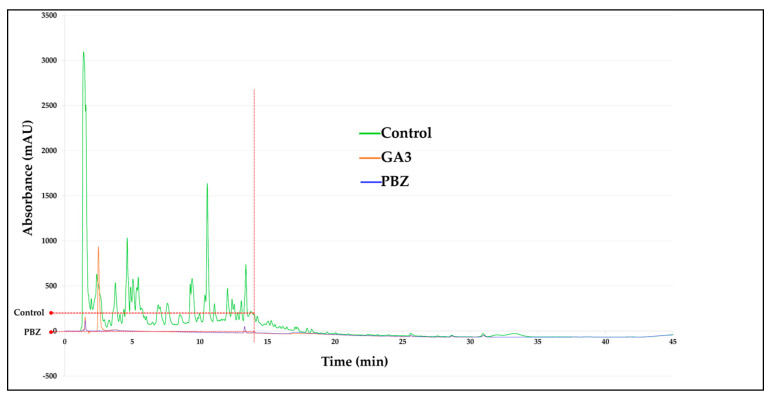
Chromatographic absorbance profile of controls (MTs without PBZ), the GA_3_ standard and PBZ-treated MTs. The GA_3_ standard (orange) shows a characteristic peak at approximately 14 min; the control (green) exhibits multiple peaks, including signals near this retention time; while the PBZ-treated sample (blue) shows no detectable peak at approximately 14 min. The vertical dashed line indicates the approximately 14 min time point, corresponding to the onset of GA_3_ expression.

In contrast, the PBZ-treated sample exhibited no detectable peak at approximately 14 min, indicating a marked reduction in or absence of compounds co-eluting with the GA_3_ standard. This finding aligns with the established function of PBZ as a potent inhibitor of GAs biosynthesis and supports the hypothesis of a substantial suppression of endogenous GAs accumulation (Figure 12).

Overall, the chromatographic profiles demonstrate a robust PBZ-induced reconfiguration of GAs-related metabolite profiles, underscoring its pronounced impact on hormonal homeostasis.

## 3. Discussion

### 3.1. Phenotypic Responses to PBZ Treatment

#### 3.1.1. MTs Development Under PBZ Treatment

The inhibition of GAs by PBZ has been widely explored as a strategy to enhance seed tuber production, promote tuber enlargement, and mitigate the inhibitory effects of high temperatures on tuberization. Previous studies have demonstrated that PBZ can increase both tuber yield and growth rate, supporting its potential as a regulator of tuber development under restrictive conditions [26].

In agreement with the phenotypic data obtained in this study, PBZ treatment resulted in reduced stem elongation and altered growth allocation patterns, characterized by a decreased leaf area and enhanced root and stolon development. This is consistent with previous studies showing that PBZ suppresses excessive shoot elongation while enhancing tuber yield and quality [16]. Accordingly, treatment with 4 mg/L of PBZ inhibited stolon elongation in in vitro MTs while promoting an increased MTs size, indicating a shift in assimilate partitioning toward storage tissue expansion.

These changes observed at the MTs level suggest that PBZ induces early metabolic and developmental reprogramming, which may persist during subsequent plant regeneration.

#### 3.1.2. Morphology of PBZ-Derived Plants

Consistent with these early alterations, PBZ-derived plants exhibited reduced stem elongation accompanied by increased stem thickness, indicating a shift from longitudinal to radial growth due to constrained cell expansion [12,26]. Additionally, plants showed an increased number of stolons and a greater root area (Figure 4), reinforcing the role of PBZ in promoting belowground organ development [17].

The reduction in leaf area, together with the compact leaf morphology, further supports restricted cell expansion and increased tissue density, as previously reported for PBZ-treated plants [13].

Since PBZ was applied exclusively during the *in vitro* MTs induction phase and not during subsequent plant growth, the phenotypic differences observed in regenerated plants likely reflect a persistent developmental reprogramming induced during early organogenesis. This suggests a potential ‘carry-over’ or physiological memory effect of PBZ exposure, whereby early hormonal perturbation of GAs biosynthesis may have long-lasting consequences on growth architecture, even in the absence of continued treatment. Similar developmental persistence following transient hormonal or environmental cues has been reported in other plant systems, supporting the concept that early metabolic and hormonal rebalancing can imprint stable growth trajectories.

### 3.2. TFs Involved in the Negative Regulation of the GA_3_-Mediated Signaling Pathway Module

Given that PBZ is a well-known inhibitor of GAs biosynthesis, its application is expected to alter GAs-mediated signaling pathways, thereby favoring the regulatory state associated with growth repression. In this context, our transcriptomics analyses identified a cluster of genes associated with the negative regulation of the GA_3_-mediated signaling pathway.

Among these, several TFs play key roles in modulating GAs responses. RGL1, a DELLA protein, acts as a negative regulator of GAs responses by repressing developmental processes [26].

GATA21/GNC further supports this interpretation. When GAs signaling is reduced and DELLA proteins accumulate, *PHYTOCHROME-INTERACTING FACTOR* (*PIF*) activity is inhibited, leading to increased expression of *GNC.* Elevated *GNC* levels repress GAs-responsive developmental processes such as germination, elongation growth, and flowering, generating phenotypes like GAs-deficient plants. Thus, *GATA21* and *GATA22* act as a transcriptional regulator that modulates GAs responses downstream of the DELLA–PIF module, functioning as a negative regulator of GAs signaling rather than directly inhibiting GAs biosynthesis [27].

*FD*, a bZIP TF involved in flowering and stem development, also supports the connection between GAs signaling and developmental regulation [28].

Similarly, *SPL3* and *SOC1* reinforce the view that GAs signaling is integrated with broader developmental programs, since these TFs form a regulatory module that integrates the photoperiod and GAs signaling to promote flowering [29]. The enrichment of these factors is consistent with the idea that PBZ treatment promotes a transcriptional environment associated with repression of GAs-dependent elongation and adjustments of developmental responses.

While up-regulated genes were primarily associated with metabolic reprogramming and stress-related adaptation, down-regulated genes provide complementary insights into the PBZ response, reflecting repression of pathways involved in active growth, cell elongation, and developmental expansion. We found eight *TRAFAC* class myosin-kinesin, essential for plant growth and development by driving intracellular transport along the cytoskeleton. This coordinated transcriptional reprogramming supports the observed phenotypic shift toward compact growth and increased storage allocation in MTs development.

### 3.3. PBZ Activation of ATH1/DELLA Proteins

*ATH1* has been proposed to exert functions that transcend simple growth repression, acting instead as an evolutionarily conserved adaptive module that enforces a compact rosette architecture as a defensive strategy against grazing, low temperatures, and drought. Through the activation of *BOP1/2* and the destabilization of *PIF* TFs, *ATH1* may coordinate environmental cues such as light and hormonal pathways like GA_3_ signaling to constrain stem elongation. The amplification of this regulatory module via *BOP1/2–TGA1/4*- and *KNAT2/6–ATH1*-positive feedback loops may point to a deeply embedded developmental program that prioritizes survival and structural resilience over elongation growth [20,30,31].

**Figure 13:**
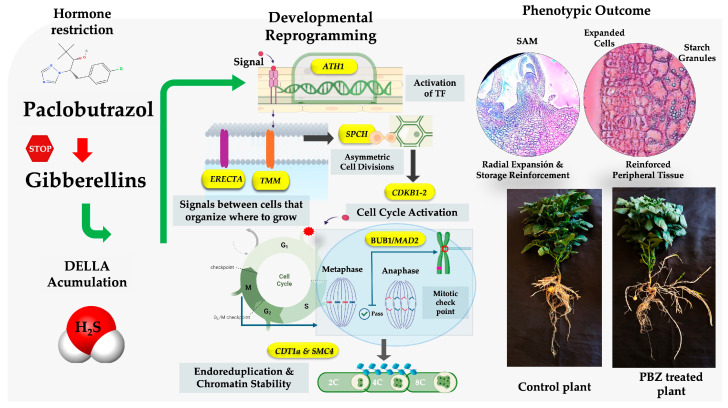
Working model summarizing the proposed hormonal, redox, and cell-cycle-associated effects of PBZ during MTs development in *S. tuberosum* L. cv. Alpha. The red arrow explains the inhibition of PBZ to gibberellins. Green arrows indicate the activation of developmental reprogramming mediated by the *ATH1* gene and the subsequent cell cycle modulation.

In *A. thaliana*, loss of *ATH1* function results in enhanced elongation, and this phenotype becomes more pronounced in global della backgrounds, suggesting that *ATH1* may act downstream of GAs signaling in coordination with DELLA proteins to restrict elongation growth [20] (Figure 13).

In addition, genome-wide analyses have identified 629 candidate *ATH1* target genes, underscoring their role as a central transcriptional regulator. Notably, 15 of these targets are represented in our PBZ-responsive network in *S. tuberosum* L. cv. Alpha, suggesting that *ATH1*-associated regulatory functions may form part of the transcription response to PBZ treatment.

Within this framework, *ATH1*-associated activation is accompanied by up-regulation of the potato ortholog of *ERECTA* (*M1BMF3*), a leucine-rich repeat receptor-like serine and threonine kinase. In *A. thaliana*, *ERECTA* has been reported to regulate aerial architecture, organ elongation, and cell proliferation [32]. Its up-regulation in potato is therefore consistent with a possible role in modulating cell division and expansion dynamics during MTs development. Importantly, ERECTA interacts with the potato ortholog of TMM (M1CSH4), TOO MANY MOUTHS, which functions as a key regulator of stomatal production in *A. thaliana* [33].

The *tmm* mutant exhibits stomatal clusters in cotyledons due to increased meristematic formation and activity, indicating that *TMM* has been reported to regulate how many cells enter the stomatal pathway and influences the balance between epidermal and stomatal cells.

TMM also interacts with SPCH (M1AIH0), SPEECHLESS, a BHLH TF that connects extracellular peptide signaling with the transcriptional regulation of asymmetric cell divisions and is required for the initiation and spacing of stomatal linages.

In *A. thaliana*, *SPCH* has been shown to integrate MAPK and brassinosteroid pathways to initiate stomatal lineage progression [34]. The presence of these components in the PBZ network may therefore suggest that PBZ treatment influences epidermal or subepidermal proliferative domains, potentially contributing to local growth patterns during MTs development. This interpretation is consistent with PBZ-responsive signaling, which extends beyond elongation control to include spatial regulation of cell proliferation.

A further point of convergence in this network is the association with cell cycle regulators. The potato ortholog of *CDKB1;2* (*M1CNC8*), cyclin-dependent kinase B1-2, has been implicated in plant cell cycle transitions, including mitosis to endoreduplication switches that are important for growth and developmental adaptation [35].

In *A. thaliana*, *CDKB1;2* promotes late stomatal lineage divisions in collaboration with *MYB124* and *MYB88* and restricts inappropriate G1 to S transitions [36]. Its up-regulation in potato is therefore consistent with controlled proliferative activity in specific developmental domains.

Likewise, the association with the potato ortholog of *CDT1a* (*M1AQW4*), a replication licensing factor, may point to a possible relationship with endoreduplication-related processes [37]. Since endoreduplication is often associated with an increased cell size and expansion of storage tissue, this interaction may be relevant to MTs enlargement.

At the chromatin level, CDT1a interacts with SMC4 (M0ZLH9), a core component of the condensin complex, which supports chromosome condensation and segregation during mitosis. In *A. thaliana*, *SMC4* is required for genome stability during cell division [38].

The incorporation of *SMC4* into the PBZ-responsive network may indicate that chromatin organization and proliferative fidelity are maintained during the developmental adjustments associated with GAs depletion (Figure 13).

These interactions support a model in which PBZ-induced reduction in GAs signaling is associated with an *ATH1*-centered regulatory response that may connect repression of elongation with localized control of proliferation, endoreduplication, and tissue patterning. Although these relationships are inferred from network structure and orthology-based functional information, they provide a plausible framework for understanding how PBZ-responsive signaling may contribute to MTs enlargement in potato.

These observations are consistent with the emerging view that transcriptional regulatory networks function as integrative hubs connecting hormonal signaling, stress adaptation, and developmental reprogramming in plants [21].

### 3.4. Chromatin Remodeling/Ribosomal Proteins and StSP6A Connection

RBL-2 (M1CMP6), an RBL protein, has been described as an integrator between cell cycle regulation and ribosomal proteins. It may play a central role in histone H3 lysine 4 (H3K4) trimethylation, regulating developmental processes such as the floral transition. It not only controls gene expression but also coordinates functional networks that integrate growth and proliferation signals. In addition, it participates as a modulator of defense and developmental responses [39,40].

RBL-2 interacts with the cell cycle through T2K12.16 (M1D0F0), the ATP synthase beta subunit which is involved in ATP production in the presence of proton gradients, indicating a link between epigenetic regulation and cellular energy status [41].

T2K12.16, in turn, interacts with the ribosome via RPS2C (M1CBF6), a conserved component involved in the correct positioning of mRNA during decoding [42,43,44].

On the other hand, CMP6 interacts with the ribosome through RPL40B (M1A5A2), which encodes a ubiquitin-fused ribosomal protein involved in ribosome biogenesis and contributes to translational efficiency and cellular growth control [45,46,47].

RPL40B interacts with RPL18 (M1BJP4), a member of the uL15 family, whose function is associated with ribosome structure and efficient translation [42]. RPL18 encodes a structural component of the 60S ribosomal subunit required for ribosome assembly and efficient translation of mRNAs into proteins [8].

The latter may be influenced by developmental factors such as *ATH1*, reinforcing the integration between developmental programs and protein synthesis. In this context, it is relevant that members of the BELL family, such as *StBEL5*, have been shown to regulate key developmental transitions, including tuberization through the activation of the tuberigen *StSP6A* [48]. Although *ATH1* has not been directly implicated in tuberigen signaling, its functional classification within the BELL family may support a broader role for these TFs in coordinating developmental reprogramming with downstream cellular processes such as translation.

Finally, RPL18 interacts with RPL11 (M1AWE0), a key ribosomal protein that forms part of the ribosomal stalk, a structure essential for the interaction of the ribosome with GTP-dependent translation factors. RPL11 has been widely recognized as a central regulator of ribosome biogenesis and cellular homeostasis, also acting as a sensor of ribosomal status and a modulator of growth and developmental processes [48,49,50,51,52]. In this context, the interaction of RPL11 with the tuberigen StSP6A may suggest an additional role in the regulation of specific developmental processes, such as tuberization.

### 3.5. Cell Cycle

PBZ is known to inhibit GAs biosynthesis, and reduced GAs signaling has been associated with restricted meristematic cell proliferation through DELLA-dependent repression of cell cycle progression [53]. In this context, the PBZ-responsive network identified in our analysis suggests that modulation of cell cycle-related pathways may form part of the developmental adjustments associated with MTs development. Reduced GAs signaling is commonly associated with lower activity of pro-proliferative regulators, including cyclins and cyclin-dependent kinases, together with increased restriction of cell cycle progression.

In parallel, PBZ may influence mitotic timing through modulation of spindle assembly checkpoint components such as BUB1 and MAD2. Although PBZ does not directly disrupt microtubules, studies using nocodazole have shown that prolonged activation of the spindle assembly checkpoint extends prometaphase and reduces post-mitotic proliferative capacity, even when mitosis is completed normally [54]. This suggests that subtle alterations in checkpoint regulation under PBZ treatment may similarly contribute to changes in proliferative behavior and developmental outcomes in MTs.

Importantly, the inhibitory effects of GAs depletion are expected to be most relevant in actively dividing meristematic cells. Once cells leave the meristematic zone, they may transition from mitotic proliferation to postmitotic growth. In *A. thaliana* roots, this transition has been associated with the onset of endoreduplication followed by rapid cell expansion [55].

In this framework, PBZ treatment may favor a shift from a predominant proliferative growth mode toward one more strongly associated with cell enlargement, which could contribute to the enhanced growth observed during MTs development.

Within the cell cycle module, we identified 26 genes associated with the microtubule motor activity and mitotic cell cycle process, 15 genes related to chromosome segregation, 12 genes involved in the regulation of cyclin-dependent protein serine/threonine kinase activity, 6 genes associated with cell cycle checkpoint signaling, and 5 genes linked to the negative regulation of mitotic cell cycle phase transition. These observations suggest that PBZ-responsive transcriptional changes extend across several levels of cell cycle control rather than affecting a single regulatory point.

Among the genes associated with negative regulation of mitotic progression, *BUB1* (*M1AMC9* and *M1D6A6*) is particularly relevant. *BUB1* encodes a serine/threonine protein kinase that functions as a core component of the spindle assembly checkpoint and localizes to the kinetochores of non-aligned chromosomes during prometaphase.

At this stage, BUB1 acts as a surveillance factor that monitors the microtubule attachment and tension at kinetochores. When attachment errors persist, BUB1 contributes to checkpoint activation and delay entry to anaphase, thereby preserving genomic stability [56]. Thus, its presence in the PBZ-responsive network is consistent with tighter regulation of mitotic progression under this treatment.

BUB1 acts together with other checkpoint effectors, including MAD2 (M1BGZ2), in the assembly of the mitotic checkpoint complex, which inhibits the anaphase-promoting complex and prevents degradation of key regulatory proteins such as cyclin B1 and securin, until checkpoint satisfaction is achieved [57].

The essential developmental role of this checkpoint is underscored by the fact that null mutants of *Mad2*, *BubR1* and *Bub3* are early embryonic lethal [58]. In this context, the enrichment of checkpoint components in our network supports the idea that PBZ treatment may be associated with reinforced control of mitotic fidelity.

Another relevant component is *MPS1-2* (*M1CU10*), a serine/threonine protein kinase that functions upstream of core spindle assembly checkpoint regulators such as *MAD2* and *BubR1*, both at the kinetochore and in the cytoplasm [59].

Proper *MPS1* activity is required for checkpoint signaling and mitotic fidelity, and perturbation of *MPS1* function in plants has been shown to impair post-embryonic development.

In *A. thaliana*, chemical genetic inhibition of *MPS1* significantly reduces lateral root formation and cell proliferation in actively growing tissues [60].

These observations are consistent with a conserved role for MPS1 in regulating proliferative capacity during plant development.

In addition to checkpoint regulators, our network includes *CDC6B* (*M1CZA2*), a homolog of the DNA replication initiation factor CDC6 and a core component of the prereplication complex. In plants, this protein plays a critical role during the early stages of DNA replication by participating in the assembly of pre-RC complexes at replication origins. However, once origins are activated, its function is no longer required for origin firing or elongation processes [59]. In *A. thaliana*, transcript accumulation peaks during early S phase, consistent with its role in replication initiation [61]. Notably, elevated levels have also been associated with the onset of endoreduplication, particularly in dark-grown hypocotyl cells, where repeated DNA replication occurs without mitosis. These findings suggest that CDC6 may participate not only in mitotic cell cycles but also in endoreduplication programs linked to post-mitotic cell enlargement [61,62].

The enrichment of spindle checkpoint regulators, replication-licensing factors, and genes associated with endoreduplication suggest that PBZ treatment is associated with reorganization of full cycle dynamics during MTs development. These transcriptional patterns support the possibility that PBZ-induced GAs depletion may contribute to a developmental shift in which restricted mitotic activity is accompanied by enhanced cellular expansion and storage organ growth.

### 3.6. Isoprenoid/Carotenoid Metabolism and ABA Biosynthesis

PBZ inhibits ent-kaurene oxidase, a cytochrome P450 enzyme involved in GAs biosynthesis downstream of ent-kaurene [23]. Consequently, carbon flux derived from GGPP is no longer directed toward GAs production and may instead be redistributed toward other isoprenoid pathways.

This metabolic reallocation has been proposed to underlie several pleiotropic effects associated with PBZ treatment, including altered stress responses and developmental processes [10].

In our transcriptome analysis, the up-regulation of *HMGR3* (*HMG3*) and *MVD2* (*M1BB88*), which encode HMG-CoA reductase 3 and diphosphomevalonate decarboxylase, respectively, contribute to the production of IPP and DMAPP, which are condensed by GGPPS1 (M1CP12), a plastidial geranylgeranyl diphosphate synthase [63] (Figure 9).

GGPPS1 catalyzes the sequential trans-condensation of three isopentenyl pyrophosphate (IPP) molecules with dimethylallyl pyrophosphate (DMAPP), resulting in the formation of geranylgeranyl pyrophosphate [64].

As GGPP levels rise, metabolic flux may be redirected toward the carotenoid pathway, where PSY1 (M1BE35), a phytoene synthase, catalyzes the first committed step of carotenogenesis by condensation of two molecules of geranylgeranyl diphosphate (GGPP) derived from the methylerythritol phosphate (MEP) pathway [65].

PSY1 interacts with carotenoid-modifying enzymes such as BETA-OHASE_1 (CHY2), and VDE1 (M1AQ52) (Figure 9).

These enzymes produce precursors of xanthophylls and violaxanthin, thereby expanding the pool of epoxy xanthophyll substrates available for oxidative cleavage by epoxycarotenoid dioxygenase (NCED) enzymes, generating xanthoxin, which enters the ABA biosynthetic pathway.

ABA2 (M1CCH5), a xanthoxin dehydrogenase, converts xanthoxin into abscisic aldehyde and subsequently into ABA [66]. These expression patterns are consistent with the enhanced metabolic pathways committed to carotenoid and ABA biosynthesis under PBZ treatment.

### 3.7. ABA and H_2_S Signaling

Elevated ABA levels can induce the activity and/or expression of L-cysteine desulfhydrases (*DES1*). The *des1* mutant has been widely used to demonstrate the importance of DES1-derived H_2_S in multiple biological processes, including autophagy and stomatal movement [67].

In this context, the ABA-related pathway of the PBZ response may be functionally connected to sulfur–L-cysteine metabolism, as suggested by the interaction network identified in our analysis (Figure 9).

One relevant component is *DLD1/LPD1-2* (*M1B1Q9*), which encodes dihydrolipoyl dehydrogenase 1 (mLPD1), a central redox enzyme of mitochondrial multienzyme complexes and reported target of protein persulfidation. mLPD1 contains reactive cysteine residues susceptible to H_2_S-mediated modification, and persulfidation may influence its redox activity and interactions within mitochondrial enzyme complexes.

Through this mechanism, H_2_S signaling could influence electron transfer efficiency and catalytic performance of mLPD1, thereby contributing to redox-dependent adjustments of mitochondrial metabolic fluxes under stress conditions [68].

Likewise, HMT3 (M1D5J8), homocysteine S-methyltransferase 3, interacts with OASA1 (CS-A), cysteine synthase 1, both of which directly participate in L-cystein biosynthesis and sulfur homeostasis, further highlighting the possible involvement of sulfur metabolism in the PBZ-responsive network (Figure 9).

OASA1 (CS-A) also interacts with MJM18.1 (M1C2W0), a pyridoxal phosphate (PLP)-dependent transferase. Since PLP serves as an essential cofactor in cysteine desulfhydrase activity, stabilizing cleavage between the α-carbon and sulfur of cysteine, this interaction is consistent with conditions that could support H_2_S production [69] (Figure 9).

In turn, the generated H_2_S has been shown to enhance ABA signaling through persulfidation of *SnRK2.6*/*OST1*, strengthening its interaction with downstream effectors such as *ABF2* and promoting ABA-dependent stomatal closure and stress tolerance.

Additional interactions with STR16 (M0ZW90), a rhodanese domain-containing sulfur-transferase associated with H_2_S metabolism, Fe–S cluster maintenance, redox balance, and ROS scavenging, further support the integration of sulfur metabolism with redox regulation in this network [70,71,72,73,74,75] (Figure 9).

Finally, the interaction between CS-A and ASP3 (M1AQQ4), an aspartate aminotransferase capable of using cysteine as an amino donor, suggests a potential link between cysteine turnover, 3-mercaptopyruvate formation and mitochondrial sulfur metabolism [76] (Figure 9).

### 3.8. Aspartate-Centered Metabolic Integration Under PBZ

The extensive connectivity of ASP3 with carbon metabolism, photosynthesis and, phenylalanine biosynthesis further suggests that it may contribute to the integration of sulfur metabolism with broader metabolic response under PBZ treatment (Figure 9).

### 3.9. Phenylalanine Metabolism

Within the phenylalanine metabolism module, the interaction network includes MHK10.21 (M1C4I0) and F27M3.9 (M1BFZ2), amine oxidases associated with the isoquinoline alkaloid biosynthesis pathway, in which dopamine is converted to 3,4-dihydroxyphenylacetic acid, a key intermediate in the morphinan alkaloid biosynthesis [77]. These specialized pathways ultimately channel L-tyrosine-derived production into alkaloid production [78].

The module also contains SDC-2 (M1B6L7 and M1ABY9), a serine decarboxylase that converts L-serine into ethanolamine, which is an essential precursor for phosphatidylethanolamine and phosphatidylcholine biosynthesis and therefore important for membrane formation and plant growth [79].

In agreement with this, the *atsdc-1* mutant displays severe developmental defects, including dwarfism, leaf necrosis, sterility, and abnormal inflorescence formation, underscoring the relevance of ethanolamine biosynthesis for normal plant development [80].

In addition, this module contains phenylalanine ammonia-lyase genes, like *PAL2* (*M1C5K7*), *PAL1* (*M1BPH2*), and related PAL proteins (M1D3Y2 and M1BY41). PAL catalyzes the first step of the phenylpropanoid pathway by converting L-phenylalanine into trans-cinnamate, and in monocots it may also function as a tyrosine ammonia-lyase [81].

In *A. thaliana*, the *pal1 pal2* double mutant accumulates phenylalanine and exhibits decreased lignin content together with altered tannin and anthocyanin biosynthesis [82], supporting the central role of PAL in phenylpropanoid metabolism, and PAL has also been implicated in defense responses against microbial pathogens, lignin and alkaloid biosynthesis, and nitrogen metabolism, and its expression is induced by pathogen infection in *Capsicum annuum* [76].

In the network ELI5 (M1BKG2), tyrosine decarboxylase 1, which is a plant enzyme involved in the biosynthesis of diverse secondary metabolites, including amides, that may reinforce the cell wall and contribute to defense against pathogen invasion, is also found [83].

Notably, H_2_S-induced accumulation of total phenols, flavonoids, and lignin has been positively associated with the expression of phenylpropanoid-related genes, including PAL, together with MYB and WRKY TFs involved in stress resistance.

Consistent with this, Huang et al. (2023) [84] showed that *CitMYB1*, *CitMYB3, CitMYB4*, and *CitWRKY23* displayed transient expression patterns characterized by an initial increase followed by a decline, whereas *CitMYB2*, *CitWRKY22*, *CitWRKY65*, and *CitWRKY72* showed up-regulation throughout storage.

### 3.10. Photosynthesis

ASP3 (M1AQQ4), which is connected with MDH1 (M1BFT7), interacts within the photosynthesis module with GAPDH (M1ATQ7), glyceraldehyde-3-phosphate dehydrogenase, a central enzyme in glycolysis and energy metabolism (Figure 9).

Beyond its metabolic role, GAPCH has been associated with plant metabolism and handling of stress responses [85]. In agreement with this, down-regulation of *GAPCp* results in severe developmental phenotypes, including arrested root growth, dwarfism, and sterility, and causes profound alterations in gene expression and in the balance of sugars and amino acids [84].

In this context, hydrogen sulfide (H_2_S) signaling has emerged as a potential integrator of photosynthesis and carbon metabolism by linking redox regulation with primary metabolic pathways. This connection originates in the chloroplast during photosynthetic sulfate assimilation, where sulfite reductase catalyzes the reduction of sulfite to H_2_S.

In *Spinacia oleracea*, H_2_S treatment was shown to up-regulate genes encoding Rubisco (*M1BQ74*) (*RBCL* and *RBCS*) and NADP–malate dehydrogenase (*NADP-MDH*) while down-regulating photorespiration-associated genes such as glycolate oxidase (*GYX*) and cytochrome oxidase (*CCO*), suggesting that H_2_S may improve carbon assimilation efficiency while reducing photorespiratory losses [86].

Consistent with this view, the proteins identified in our network are mainly associated with central metabolic routes, including glycolysis, the tricarboxylic acid (TCA) cycle, the pentose phosphate pathway, and redox homeostasis, and nitrogen and sulfur assimilation [87]. In addition, several proteins that were identified, like GAPDH, ribulose-1,5-bisphosphate carboxylase/oxygenase (RuBisCO), phosphoribulokinase (PRK), and glutamine synthetase (GS), have been previously described as redox-sensitive enzymes and potential targets of H_2_S-mediated regulation [88].

Together, these observations support the interpretation that H_2_S-related signaling contributes to the coordination of redox balance and metabolic adaptation under PBZ treatment.

Additionally, in a previous study, PBZ-treated plants showed increased chlorophyll during rosette development [89]. This is consistent with the activation of 12 chlorophyll-related genes in the transcriptome, such as *Cab1*, *Lhcb1-2*, *LHCB2.1* (*M0ZYW1*), *LHCB4.1* (*M1AFT5*), *LHCB6* (*M1AY18* and *M1AY19*), *LHCA2* (*M1B545*), *PORB* (*M1B8N6*), *LHCA1* (*M1C5L1*), *PORA* (*M1CC88*), *LHCB5* (*M1CIH8*) and *LHCB1.3* (*M1DQW0*).

### 3.11. Carbon Metabolism

ASP3 (M1AQQ4) is connected to the carbon metabolism module to PDC1, NADP-ME4, PCKA, PPC3 and MDH1 (Figure 9), suggesting that PBZ-responsive genes are associated with several core pathways of primary metabolism. PDC1 (M1A6Z4), pyruvate decarboxylase 1, has been implicated in ethanolic fermentation under low oxygen conditions, and its overexpression enhances tolerance to hypoxia, whereas the *pdc1* mutant shows increased sensitivity to anoxia, underscoring its importance for survival under oxygen-limiting environments [90].

NADP-ME4 (M1CYF9), a chloroplastic NADP-dependent malic enzyme, contributes to the generation of NADPH from malate and thereby supports ROS-related defense responses and biosynthetic processes such as lignin deposition.

In *A. thaliana*, reduced *NADP-ME* activity is associated with lower ROS accumulation and increased susceptibility to pathogens, such as *Colletotrichum higginsianum* and *Pseudomonas syringae*, highlighting its contribution in basal resistance and cell wall-associated defense response [91]. This enzyme family has also been associated with routes that reduce photorespiratory loss [92].

*PCKA* (*M1A7J1*), phosphoenolpyruvate carboxykinase, is responsible for catalyzing the transformation of oxaloacetate into phosphoenolpyruvate during the gluconeogenic pathway and has been linked to sucrose supply, pH regulation, and amino acid metabolism during early seedling development [93].

*PPC3*, phosphoenolpyruvate carboxylase 3, contributes to oxaloacetate production in the tricarboxylic acid cycle, and reduced PEPC activity has been shown to disrupt the carbon–nitrogen balance and decreased malate and citrate synthesis and promotes starch and sucrose accumulation [94].

On the other hand, PBZ induces stress-like physiological responses that may be mediated by a reprogramming of the translatome, favoring the translation of genes associated with energy metabolism and stress adaptation over those involved in growth and stress [90,94].

In our case, these observations are consistent with our transcriptomic data, and metabolic shift is supported by the expression of genes such as *M1BFT7* (malate dehydrogenase) and *M1B824* (malate synthase), which are involved in central carbon metabolism. Additionally, *M1DDR8* (UDP-glucose 6-dehydrogenase) and *M1BX36* (glycosyl transferase) suggest active carbohydrate remodeling, reinforcing the idea that PBZ promotes metabolic adjustments linked to stress resilience and resource reallocation [95].

Histological analysis further supports this interpretation, as PBZ-derived MTs exhibited a higher accumulation of starch granules compared to control samples (Figure 3). This increase in starch deposition provides direct anatomical evidence of enhanced carbon allocation toward storage compounds and suggests an early activation of tuberization-related metabolic pathways [96].

### 3.12. Model of PBZ Reprograming of MTs Development

To integrate the molecular and physiological responses associated with PBZ-induced MTs development, Figure 13 summarizes a working model in which PBZ inhibits GAs biosynthesis, therefore favoring DELLA accumulation. In this framework, DELLA is proposed to contribute to MTs development through two coordinated but interconnected processes. First, reduced GAs signaling restricts elongation growth and, together with *ATH1*-associated developmental reprogramming, is linked to the transition from stolon-like growth to a more compact developmental state [18,97]. Regulatory components, such as *ERECTA*, *TMM*, and *SPCH*, may contribute to local patterning of cell proliferation and asymmetric cell division, as well as with downstream modulation of cell cycle regulators such as CDKB1-2.

Second, PBZ treatment is associated with a redox adjustment that may favor endogenous H_2_S-related signaling. As a redox mediator, H_2_S has been shown to modulate protein activity through persulfidation and may influence cell cycle progression, thereby contributing to balance among proliferation, checkpoint control and endoreduplication required for radial expansion [98]. In parallel, the association of CDT1a and SMC4 with this network is consistent with the maintenance chromatin stability during this developmental transition. These coordinated responses offer a plausible framework to explain the phenotypic response to PBZ, including reduced elongation, reinforced peripheral tissues, increased starch accumulation, and radial enlargement of the developing MTs.

## 4. Materials and Methods

### 4.1. Plant Material

#### 4.1.1. Potato Shoot Micropropagation

The micropropagation of potato plantlets was performed using Murashige and Skoog (MS) medium [99] supplemented with 10 g/L of sucrose, 3 g/L of activated charcoal (CAT: 242276, Sigma-Aldrich, St. Louis, MO, USA), and 3 g/L of Gelrite (GELZAN, CAT: G1910, Sigma-Aldrich, St. Louis, MO, USA).

The medium was adjusted to pH 5.8 prior to autoclaving. After inoculation, the explants were incubated in a growth chamber (PERCIVAL, Perry, IA, USA) at 25/17 °C under fluorescent light at 25 µmol m^−2^ s^−1^ of irradiance for a total period of 3 weeks.

Micropropagation jars were sealed with a transparent plastic membrane to allow for gas exchange between the internal atmosphere and the external environment.

#### 4.1.2. Potato MTs Induction

Potato MTs induction protocol was made according to Valencia et al. 2022 [6]. Stolon explants of approximately 3 cm in length were cultured in MR8G62iP medium: MS medium supplemented with 10 mg/L of 2iP, 8% sucrose, 6 g/L of gelrite, 3 g/L of activated charcoal, pH 5.8, an osmotic potential of 2.02 mPa and the corresponding concentration of PBZ (0, 0.25, 1, or 4 mg/L).

All treatments were incubated under continuous darkness and temperature cycles of 22 °C for 8 h and 17 °C for 16 h. Darkness was ensured by wrapping the culture jars in aluminum foil followed by black polyethylene bags. As controls, stolon explants were cultured in MR8G62iP [6].

After MTs development under PBZ treatment, plant regeneration was performed by subculturing MTs to MS medium supplemented with 1% sucrose and 3 g/L of gelrite and incubated at 25 ± 2 °C under a 12/12 h photoperiod at 50 µmol/m^2^-s irradiance provided by fluorescent lamps (T8 Phillips P32T8/TL850, Eindhoven, The Netherlands). Regenerated plants were transferred to soil and grown under controlled greenhouse conditions for 4 months. No PBZ was applied during *ex vitro* growth; thus, all observed phenotypic traits reflect the developmental effect of prior in vitro PBZ exposure.

#### 4.1.3. Histological Analysis of MTs

Randomly chosen MTs induced in MR8G62iP and PBZ (4 mg/L) were collected and fixed in FAE (5% formaldehyde, 10% acetic acid, and 50% ethanol), followed by dehydration in a series of ethanol dilutions (20%, 40%, 60%, 80% and 100% ethanol) for 2 h each according to Valencia-Lozano et al. 2021 [100]. Samples were embedded in Technovit 7100 (Heraeus Kulzer, Wehrheim, Germany) according to the manufacturer’s instructions. Sections (14 μm) were obtained on a rotary microtome (Reichert-Jung 2040; Leica, Nussloch, Germany). Tissue sections were stained with a 0.02% Toluidine Blue solution (HYCEL, Zapopan, Mexico), and samples were stained for 3 min, washed with distilled water for 1 min, and air-dried. Pictures were taken using a DM6000B microscope (Leica, Wetzlar, Germany) [100].

Starch granules were quantified from histological sections of MTs. Images were divided into four quadrants, and starch granules were counted in 28 cells per quadrant, resulting in 112 analyzed cells per treatment. Statistical differences between control and PBZ-treated samples were evaluated using Welch’s *t*-test.

### 4.2. RNA Extraction and Library Preparation for Transcriptome Sequencing

For transcriptomic analysis, the 4 mg/L PBZ treatment was selected based on its strongest and most consistent phenotypic response in MTs development, including maximal tuber enlargement and clear inhibition of stolon elongation without inducing tissue damage. This condition was therefore considered the most representative of the PBZ-induced developmental reprogramming and was used for RNA-seq analysis. Lower PBZ concentrations (0.25 and 1 mg/L) were excluded from RNA-seq due to their reduced effect size and higher phenotypic variability, which could compromise detection of robust transcriptional changes.

Total RNA isolation was performed using the Trizol reagent (Invitrogen, Carlsbad, CA, USA). The RNA concentration was quantified spectrophotometrically at 260 nm, and purity was verified by assessing the 260 nm/280 nm absorbance ratio. RNA integrity was confirmed via electrophoresis on a 1% (*w*/*v*) agarose gel.

Library preparation and transcriptome sequencing were conducted by GENEWIZ (Plainfield, NJ, USA) using an Illumina HiSeq 2500 platform (Illumina, San Diego, CA, USA). Raw reads were evaluated for quality using FastQC (http://www.bioinformatics.babraham.ac.uk/projects/fastqc/ accessed on 24 November 2025) and subsequently processed to remove adapter sequences and low-quality bases with Trimmomatic [101]. Filtered reads were aligned to the *S. tuberosum* L. reference genome available in Phytozome v12.1 using the STAR aligner (v2.5.2b) [102], resulting in BAM (Binary Alignment/Map) files. Gene-level read counts and transcript quantification were obtained using the featureCounts function from the Subread package (v1.5.2) [103].

### 4.3. Quantification of Gene Expression Levels and Differential Expression Analysis

RNA-seq analysis was performed using three independent biological replicates for each experimental condition (control and PBZ treatment), resulting in a total of six libraries. Raw sequencing reads were quality filtered and mapped to the *S. tuberosum* reference genome. Gene-level raw counts generated using featureCounts were used for differential expression analysis with DESeq2 v1.12.4 using the design formula ~ treatment. The Benjamini–Hochberg method was applied for false discovery rate correction, and genes with adjusted *p*-values (FDR < 0.05) and |log2FoldChange| ≥ 0.5 were considered significantly differentially expressed. FPKM values were calculated exclusively for expression visualization and heatmap representation. Both up- and down-regulated gene sets were subjected to GO enrichment and functional interpretation; however, pathway representation was considered separately to capture both activation and repression patterns.

### 4.4. PPI Network Analysis of MTs Development with PBZ

A gene network with high confidence (0.500) was performed with the STRING database v12.0 [104] based on *S. tuberosum* L., homologous genes present in the *S. tuberosum* genome in the Sol genomics network [105]. A gene identifier (Id) was made according to the UNIPROT [106] and NCBI database [107].

Homologs in *S. tuberosum* L. that were greater than 60% in protein sequence compared with *A. thaliana* were considered. Oligonucleotides were designed by qPCR (2^−∆∆CT^ method analysis) [108].

### 4.5. Validation of the Transcriptome Analysis

RNA isolation and cDNA synthesis were carried out, and subsequent amplification was performed using the SYBR™ Green Master Mix (ThermoFisher, Waltham, MA, USA) in a Real-Time PCR System (CFX96 BioRad, Hercules, CA, USA). The RT-qPCR validation was designed as a targeted approach focusing on representative genes involved in key regulatory pathways identified in the RNA-seq analysis, rather than a comprehensive validation of all differentially expressed genes. Future validation will include representative down-regulated genes associated with growth-related pathways.

Eight genes were chosen to validate the transcriptome analysis of potato MTs development under PBZ treatment: *GGPP* (*M1CP12*), geranylgeranyl pyrophosphate synthase; *ERECTA* (*M1BMF3*), a leucine-rich repeat receptor-like serine/threonine kinase; *ABA2* (*M1CCH5*), Xanthoxin dehydrogenase; *OASA1* (*CS-A*) cysteine synthase 1; *ASP3* (*M1AQQ4*), aspartate aminotransferase 3; *SMC4* (*M0ZLH9*), a core component of the condensin complex; *ATH1* (*M1CXR7*), *ARABIDOPSIS THALIANA HOMEOBOX GENE 1*; and *CDT1a* (*M1AQW4*), a replication licensing factor (Table S3).

The genes *EF1* and *SEC3* were used as housekeeping reference genes and as normalizers. Each sample was analyzed using five biological replicates, with each qPCR reaction performed in technical triplicate. Relative gene expression levels were calculated using the 2^−ΔΔCt^ method [108].

### 4.6. Metabolite Quantification by HPLC

Potato tubers from each treatment were collected and lyophilized in 50 mL Falcon tubes before being pulverized using a mortar and pestle.

A 115 mg aliquot of the resulting powder was suspended in two equal volumes of a solution comprising 1:1 acetonitrile and H_2_O.

The mixture underwent three rounds of centrifugation at 10,000 rpm for 20 min each, with the supernatant recovered after each cycle.

The collected liquid sample was then concentrated in a SpeedVac for 3 h, and 200 µL of the concentrate was transferred to an HPLC vial.

Finally, HPLC analysis was performed for each metabolite of interest, using its respective standard for peak identification [109].

## 5. Conclusions

PBZ-induced inhibition of GAs biosynthesis was associated with extensive transcriptional and metabolic reprogramming during potato MTs development. Transcriptomic analyses suggest that reduced GA signaling may promote coordinated activation of stress-responsive, hormonal, and developmental regulatory pathways linked to metabolic adaptation and storage organ formation.

The enrichment of ATH1/DELLA-associated regulatory components, together with genes related to cell cycle control, chromatin organization, sulfur metabolism, and carbon allocation, supports a model in which PBZ treatment may shift developmental programs from elongation-associated growth toward cellular expansion, metabolic adjustments, and storage-related processes.

In addition, histological observations showing increased starch granule accumulation in PBZ-treated MTs are consistent with enhanced carbon partitioning toward storage metabolism. Collectively, these findings provide a plausible framework for understanding how PBZ-responsive signaling networks may contribute to MTs enlargement and developmental adaptation in potato.

## Figures and Tables

**Table 1:** Phenotypic characterization of control and PBZ-treated (4 mg/L) potato plants. Values represent the mean ± SD obtained from three independent biological replicates. PBZ-treated plants exhibited reduced stem and root length, whereas stem width, root area, and stolon number showed higher mean values compared with control plants. Quantitative measurements were obtained from triplicate evaluations performed over a four-month growth period.

Treatment	Stem Length (cm)	Stem Width (cm)	Root Length (cm)	Root Area (cm^2^)	Leaf Area (cm^2^)	Number of Stolons
**Control**	22.7 ± 2.78	1.186 ± 0.286	28.03 ± 9.86	102.2 ± 29.62	260.4 ± 27.65	8 ± 0.57
**PBZ**	20 ± 0.232	1.285 ± 0.488	20.71 ± 0.99	103.4 ± 1.87	197.2 ± 0.11	12 ± 1.41

## Data Availability

The dataset presented in this study can be found in NCBI BioProject online repository with the accesion number PRJNA1456097, and can be found below: https://www.ncbi.nlm.nih.gov/bioproject/1456097, accessed on 16 May 2026.

## Supplementary Materials

The following supporting information can be downloaded at: https://www.mdpi.com/article/10.3390/ijms27104618/s1.

## Abbreviations

GAs	Gibberellins
GA_3_	Gibberellic acid
H_2_S	Hydrogen sulfide
MTs	Microtubers
PBZ	Paclobutrazol
TF	Transcription factor
TFs	Transcription factors
PPI	Protein–protein interaction
