# Supplementary material for: Transcriptomic Insights into Paclobutrazol-Induced Modulation of Metabolic and Signaling Pathways During Microtuberization of Potato Solanum tuberosum L
Source: Int J Mol Sci. 2026 May 21;27(10):4618. doi: 10.3390/ijms27104618 (PMC13207467; doi:10.3390/ijms27104618)
Supplement: Supplementary file 1 [file ijms-27-04618-s001.zip › Supplementary Figure S1.pdf]

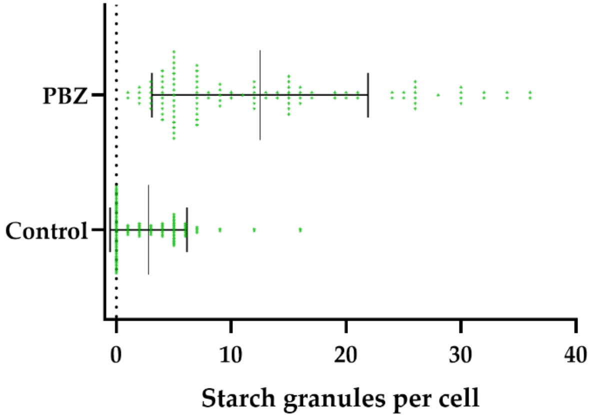

Supplementary Figure S1. Quantification of starch granules per cell in histological sections of control and PBZ-treated microtubers. Data represent 112 analyzed cells per treatment. Values are shown as mean ± SD. Statistical significance was determined using Welch's t-test (**** $p < 0.0001$).
